# Comparison of superficial wound documentation using 2D forensic photography, 3D photogrammetry, Botscan© and VR with real-life examination

**DOI:** 10.1007/s12024-021-00393-x

**Published:** 2021-08-18

**Authors:** Federico Massini, Lars Ebert, Garyfalia Ampanozi, Sabine Franckenberg, Lena Benz, Till Sieberth

**Affiliations:** 1 University of Zurich, 8057 Zurich, Switzerland; 2 Institute of Forensic Medicine, University of Zurich, 8057 Zurich, Switzerland; 3 3D Centre Zürich, University of Zurich, 8057 Zurich, Switzerland; 4 Institute of Diagnostic and Interventional Radiology, University Hospital of Zürich, 8091 Zürich, Switzerland; 5 University of Bern, 3012 Bern, Switzerland

**Keywords:** Forensics, Photogrammetry, 3D Reconstruction, Photobox, Clinical examination

## Abstract

Evidence acquisition, interpretation and preservation are essential parts of forensic case work that make a standardized documentation process fundamental. The most commonly used method for the documentation and interpretation of superficial wounds is a combination of two modalities: two-dimensional (2D) photography for evidence preservation and real-life examination for wound analysis. As technologies continue to develop, 2D photography is being enhanced with three-dimensional (3D) documentation technology. In our study, we compared the real-life examination of superficial wounds using four different technical documentation and visualization methods.

To test the different methods, a mannequin was equipped with several injury stickers, and then the different methods were applied. A total of 42 artificial injury stickers were documented in regard to *orientation*, *form*, *color*, *size*, *wound borders*, *wound corners* and *suspected mechanism of injury* for the injury mechanism. As the gold standard, superficial wounds were visually examined by two board-certified forensic pathologists directly on the mannequin. These results were compared to an examination using standard 2D forensic photography; 2D photography using the multicamera system Botscan©, which included predefined viewing positions all around the body; and 3D photogrammetric reconstruction based on images visualized both on screen and in a virtual reality (VR) using a head-mounted display (HMD).

The results of the gold standard examination showed that the two forensic pathologists had an inter-reader agreement ranging from 69% for the *orientation* and 11% for the *size* of the wounds. A substantial portion of the direct visual documentation showed only a partial overlap, especially for the items of *size* and *color*, thereby prohibiting the statistical comparison of these two items. A forest plot analysis of the remaining six items showed no significant difference between the methods. We found that among the forensic pathologists, there was high variability regarding the vocabulary used for the description of wound morphology, which complicated the exact comparison of the two documentations of the same wound.

There were no significant differences for any of the four methods compared to the gold standard, thereby challenging the role of real-life examination and 2D photography as the most reliable documentation approaches. Further studies with real injuries are necessary to support our evaluation that technical examination methods involving multicamera systems and 3D visualization for whole-body examination might be a valid alternative in future forensic documentation.

**Supplementary information:**

The online version contains supplementary material available at 10.1007/s12024-021-00393-x.

## Introduction

The objective documentation and description of superficial injuries is a fundamental part of forensic examinations [1]. The documentation process is often standardized and includes a collection of written statements together with drawn sketches of the injury locations and photographic documentation of the evidence [2]. Such documentation preserves the forensic evidence and should be in a format that enables specialists to form a second opinion, which would ideal confirm the original conclusions and thus allow rigorous quality control for the prevention of misdiagnoses [3]. To characterize superficial injuries, information about their localization, color, size, form, orientation, wound angles and corners, as well as the suspected mechanism of injury involved, must be documented [1]. Currently, the established method for the documentation of this forensic information is two-dimensional (2D) photography [4], including written findings of the injuries made by a forensic pathologist. Since the replacement of analogue cameras with digital cameras, professional photographers and forensic pathologists, medical examiners and specialists with police forces can perform photo-documentation. Experience has shown that it is not enough to rely on the automatic functions of the digital camera to meet the requirements of forensic documentation and that proper training is required prior to photo-documentation [5]. Even if a 2D photograph has no technical flaws, it still consists of a projection of a three-dimensional (3D) scene on a 2D plane and therefore cannot preserve all the spatial information of a superficial injury [4]. One way to avoid such a loss of information is to use 3D photogrammetry. With this method, it is possible to document the 3D shape and orientation of an object in space by using two or more overlapping images [6]. In forensic medicine, photogrammetry has been used to match injury-causing objects to detected wounds [7–10] and has been shown to be more precise in the measurement of wound size than the 2D photography method [11]. 3D documentation requires the examined object to stay stationary during data acquisition, which can be an issue, especially with living persons. A solution to this issue has been presented by Leipner et al., who used a multicamera device to perform 3D documentation on living people [12]. However, the final 3D model is usually visualized on a 2D computer screen, thereby eliminating depth information. One possibility for visualizing 3D injuries in 3D is through virtual reality (VR) head-mounted displays (HMDs) [4]. In addition to the advantage of observing 3D models in 3D, it is also possible to interact with and modify the models in this format [13, 14]. In a previous work, Koller et al. compared the accuracy of injury size measurements in 2D forensic photographs and with a 3D photogrammetric reconstruction in VR. It was shown that the VR measurements were more accurate than the measurements obtained using conventional 2D forensic photographs but less accurate than the measurements obtained using a 3D model visualized on a 2D screen [15]. However, previous studies have only measured the dimension of the injuries and have not analyzed whether it is possible to medically assess the acquired 3D documented injuries with the multi-camera device known as Botscan© [12].

Previous studies have been limited to objective measurements only. In this article, we compare the real-life examination and interpretation of injuries with technically assisted methods using standard 2D forensic photography, 2D photography with the Botscan© multicamera system and 3D photogrammetrically reconstructed 3D models examined both on a screen and in VR using an HMD.

## Methods

As the aim of the current study was to analyze the forensic examination of superficial injuries using several visualization methods, we first established a gold standard based on real-life examination by board-certified forensic pathologists. For this study, two board-certified forensic pathologists each carried out an examination. The results of these direct examinations were then compared with those obtained using the four abovementioned technical documentation and display methods. While the 2D forensic photographs were taken separately, according to forensic standards, the other display methods relied on the same photographs taken by the multicamera device known as Botscan©. This documentation setting consisted of 70 synchronized DSLR cameras positioned around a person who was standing in an upright position that remotely and simultaneously took 70 pictures [12]. These photographs were then used for the generation of the 3D models that were subsequently analyzed both on screen and in VR.

## Materials and hardware

For the real-life documentation of the injuries, we used the same mannequin with injury stickers, 2D forensic photographs and 3D models as those previously used by Michienzi et al. [11]. We acquired a second set of 2D photographs using Botscan©V1.0 (Botspot GmbH, Berlin, Germany), which is also known as Photobox. This separate dataset was acquired with additional scale bar stickers attached next to the injuries. The dataset was then used for examination using only the 2D photographs acquired by Photobox, without any further processing by a 3D model. The Photobox photos and the 2D photographs provided by Michienzi et al. were displayed as-is on a standard office computer screen, whereas the 3D models required dedicated additional processing.

**Fig. 1:**
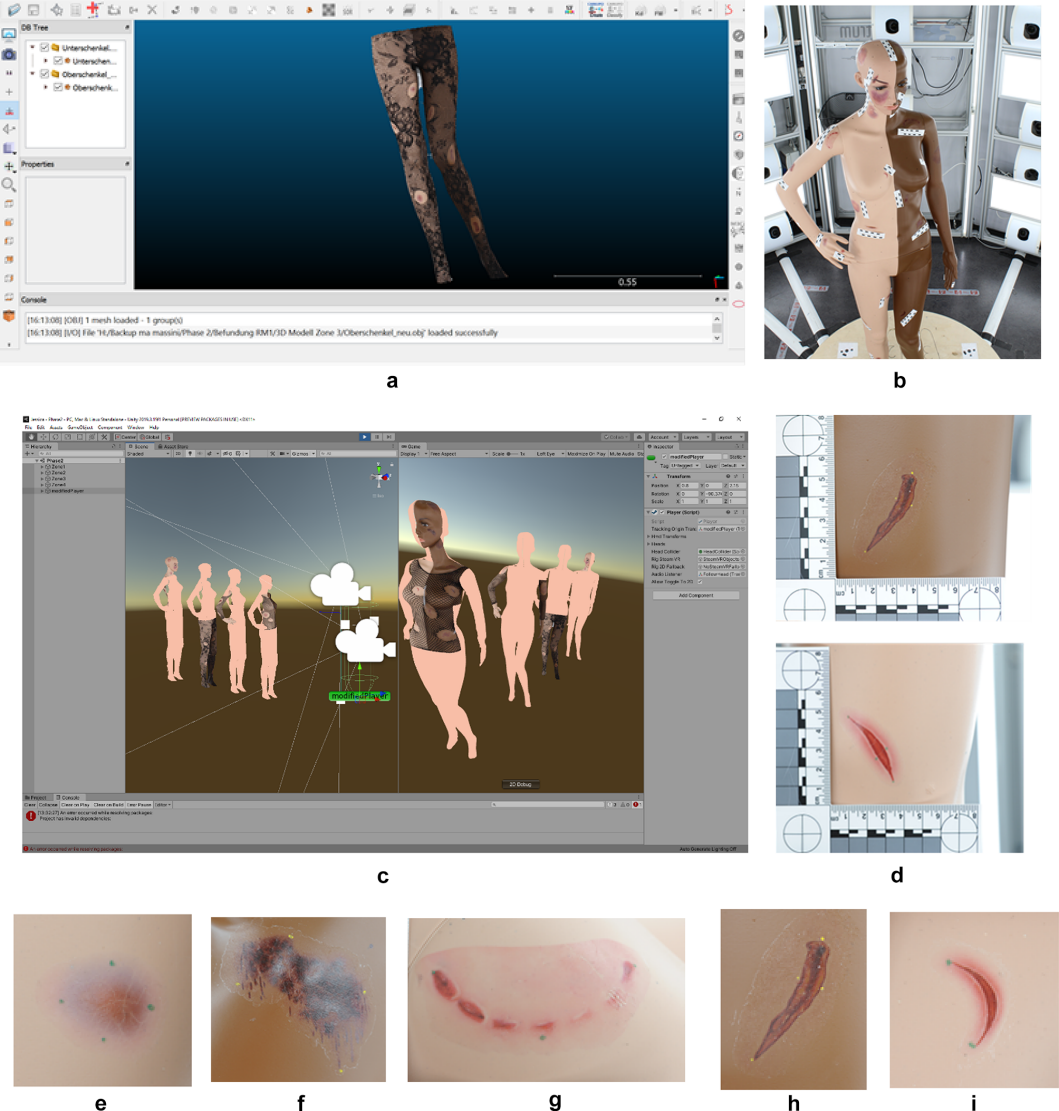
Samples from the image collection of the different image devices and a summary of the different types of wound stickers. **a**) Screenshot of one documented zone using the 3D photogrammetry model on the screen. **b**) One of the top anterior photographs taken from the Photobox by Botscan©. **c**) Screenshot of the four VR models used; for the documentation, only one model at a time was displayed. **d**) 2D photographs of dark and light skin. **e**-**i**) Types of wound stickers used; from left to right: haematoma, abrasion, bite wound, deep cut, and superficial cut

For the visualization of the 3D models on a 2D computer screen, the software known as Cloud Compare (Version 2.6.1) was used. This software allows the rotation, scaling and panning of objects, which means that all the injuries on the 3D model could be viewed properly. The VR examination was performed using an HTC Vive (HTC, Taoyuan, Taiwan) HMD with two controllers and in Unity (Version 2018.1.8f1 Personal, Unity Technologies, San Francisco, United States) using Steam VR (v1.2.3, Valve Cooperation, Bellevue, Washington, United States). Due to the interactive nature of VR, the examiners were able to walk around the mannequin and move closer to the injuries they examined. Together with the gold standard, the four methods we compared in the current study (outlined in Fig. 1) are as follows:

- Two real-life examinations of the mannequin
- Standard forensic 2D photographs with scale bars (Fig. 1d)
- Photobox data:
  - 2D photographs from 70 predefined viewpoints from all around the body, with scale bar stickers next to the injuries (Fig. 1b)
  - 3D model based on 70 photographs obtained from predefined viewpoints
    - Visualized on a computer screen (Fig. 1a)
    - Visualized in a VR HMD (Fig. 1c)

## Examination

The 42 injuries on the mannequin were documented in writing by two board-certified forensic pathologists. These results were then compared with those obtained from examinations that used the four different indirect display methods. This method-specific analysis was performed by resident forensic pathologists. We were able to recruit four forensic pathology residents with 20, 17, 8, and 5 months of experience in forensic medicine. One of the board-certified pathologists used for the real-life documentation of the mannequin’s injuries had 14 years of working experience in forensic pathology, while the other pathologist had 5 years of experience. To avoid personal bias and ability influence on the methods, the 42 injuries were subdivided into four groups, each one of which focused on different body zonesand contained between ten and eleven wounds (Fig. 1e-i, Table 1).

**Table 1:** Summary of the zone’s divisions

Zone 1	Anterior front of the torso and anterior left part of the head
Zone 2	Both upper extremities
Zone 3	Both lower extremities and lower abdomen
Zone 4	Posterior front of the torso, anterior right, and dorsal area of the head

Each zone was then analyzed with a different display method. By interchanging the zones regarding the depiction methods, we ensured that each resident would analyze every zone with a different display method and that no single zone would be analyzed with the same method by two different residents. To make the orientation in VR easier for the pathologists, the different zones were combined with a solid color representation of the whole-body context. The 2D Photobox analysis was performed last with each pathologist, as the images taken by Photobox showed large proportions of the body and thus required large amounts of image manipulation to avoid detection bias. Additionally, the injury dimension measurements were not performed using the 3D model on the screen, as was performed previously, proving that both methods allow for a higher level of accuracy than that allowed for by 2D forensic photographs [10, 11, 15].

Each injury was examined regarding *orientation*, *form*, *color*, *size*, *wound borders*, *wound corners* and *suspected mechanism of injury*. These parameters were chosen in accordance with the documentation routine in forensic medicine. We chose to implement the variable of wound size to assess differences depending on the wound morphology or the underlining skin tone. A summary of the description items used is presented in Table 2.

**Table 2:** Summary of the categories used for the examination

Orientation	In line with body/leg/arm axis; horizontal to body/leg/arm axis; top left bottom right; top right bottom left; top outside bottom inside; top inside bottom outside; other orientation
Form	Oval; round; grouped; parallel; striped; linear; elongated; curved; cloud-shaped; point-shaped; geometric; map-shaped; spindle-shaped; flat
Colour	Grey; blue; yellow; orange; red; brown; central paled; dark red; dark brown; violet; livid; brown–red; blue-red
Size	Eye examination with a ruler (mm) for maximal length and width; if seen as round, only one number for the diameter (mm); eventually, an assessment for the depth of the injury was also communicated
Wound borders	Sharp, regular; unsharp, irregular; skin reddening; skin abrasion; skin under-bleeding; not assessable
Wound corners	Acute; blunt; odd; even; with extension; not assessable
Mechanism of injury	Sharp violence (cut, stab, cut/stab combination); semi-sharp violence, e.g., bite wound; blunt violence (skin under-bleeding, skin abrasion, laceration, contused lacerated); thermic violence

Additionally, the physicians had the opportunity to give feedback about the subjective positive and negative aspects of every display method, as well as how sure they felt while performing the examination using each display method.

## Data analysis

We compared the results of the real-life examinations of the mannequin with those from the four different display methods; we rated them to as *different from*, *similar to* or *identical to* each other. We rated two findings as *different* if there was no consensus. A partial consensus was rated as *similar*, and a complete overlap was rated as *identical*. For example, if a wound was characterized as having sharp borders with skin abrasion in both real-life examinations, then we rated the examinations as *identical* in regard to the category of the wound corners. If the same wound was characterized as having sharp borders but no skin abrasions using the display in VR, we classified this examination as being *similar* to the gold standard (Table 3). For comparisons between the display methods and the following statistical analysis, we used only the *identical* items from the two direct examinations because we wanted to have a consensus for our gold standard as a comparison base.

**Table 3:** Summary of the criteria used for the classification of *similar* per assessed item, with examples on the right-hand side

Orientation	Every description that resulted in the same orientation, even if they had different wording	"In-the-arm axis" and "approximately-in-the-arm axis"
Form	Curved forms were grouped together as being similar, as opposed to square-shaped, and vice versa	"round" and "oval", "linear" and "striped"
Colour	An overlap in the colour description	"red to purple" and "red"
Size	We defined all size differences up to 1 cm as *similar*	"measuring 2.5 × 2.5 cm" and "measuring 2.4 × 2.8 cm"
Wound borders, Wound corners, Mechanism of injury	For the characterization of these three items, a main group and a possible subcategorization were used, as in forensics. We defined similarities are being between two items that had the same main group, even though one of the items may have been missing a subcategorization	"blunt violence with skin under-bleeding and lacerations" and "blunt violence with lacerations", "sharp wound borders with skin under-bleeding" and "sharp wound borders with skin reddening"

The items related to *form* were described with great variation in the terminology. To allow a statistical analysis, we decided to merge some terms if their differences were not significant, as follows:

- Round and oval
- Cloud-shaped and map-like
- Spindle, elongated, linear, and striped

We performed an analysis to evaluate whether the examinations that used the technical display methods were significantly different from those that used the gold standard. We compared all the categories from the wounds that were examined identically in the direct documentation of the mannequin with the examination of the corresponding wounds using each display technique. The statistical analysis was performed by the Department of Biostatistics of the University using R (Version 4.0.2, R Foundation for Statistical Computing, Vienna, Austria). A copy of the code used and the resulting forest plots can be found in the Appendix.

## Results

The concordance in the real-life documentation varied substantially between the documented categories. The highest inter-reader agreement was 69% for the orientation category, whereas the lowest inter-reader agreement was 11% for the size of the wounds. Because we defined two sizes as being *similar* up to a difference of one centimeter, 81% of the measured wounds ended up in this classification. The three categories of orientation, form and color each had 29% of their items documented differently. The direct documentation of the mannequin was also rated differently in regard to the subjective certainty that the forensic pathologists felt during the examination. For both board-certified forensic pathologists, one rated their examination confidence with an 8, while the other rated their confidence as a 3, on a scale of 1 (uncertain) to 10 (most certain). Both pathologists felt that they missed the three-dimensional shape of the injury due to the flat stickers.

For the technical display methods, the categories of *size* and *color* were omitted due to the large differences already found in the gold standard method. Apart from those categories, all the techniques had their lowest agreement values for the suspected mechanism of injury, which ranged from 36% using the Botscan© technique to 23% using VR. The highest concordance value was 83% for the wound borders obtained using 2D forensic photography. All four residents were more comfortable with 2D forensic photography and the 2D Botscan© technique compared to 3D photogrammetry both on screen and using the VR approach. However, one out of the four residents preferred VR over 3D photogrammetry on screen. The total time required for all four examination procedures ranged between 1.25 and 2 h (Table 4 and 5).

**Table 4:** The proportion in the three categories of *identical*, *similar*, and *different* are given in percentages, while the corresponding absolute number is given in parenthesis. The low values for the identically examined wounds in the categories of color and size made a statistical analysis futile and were therefore not presented

Direct Wound examination on Mannequin					
Form	Colour	Size	Wound borders	Wound corners	Mechanism of injury
50 (21)	19 (8)	11 (5)	43 (18)	67 (28)	52 (22)
21 (9)	52 (22)	81 (34)	50 (21)	17 (7)	43 (18)
29 (12)	29 (12)	7 (3)	7 (3)	17 (7)	5 (2)
Subjective level of certainty			8	3

**Table 5:** We used for the comparison of the technical display methods only the items that were documented identically in the direct wound documentation. Because of their changing amounts, only the absolute number of identical examinations is shown. For the technical display methods, the four forensic physicians ranked the used methods on a scale of 1 to 4, with 1 being defined as the most certain method

Technical display methods					
	Orientation	Form	Wound borders	Wound corners	Mechanism of injury
2D forensic photography	21	17	15	19	6
Botscan© (Photobox)	15	13	13	13	8
3D photogrammetry	22	16	11	22	6
VR	20	17	7	20	5
Subjective order of certainty by physician					
2D forensic photography	1, 1, 1, 1		Botscan© (Photobox)	2, 2, 2, 2	
3D photogrammetry	3, 4, 3, 3		VR	4, 3, 4, 4	

**Fig. 2:**
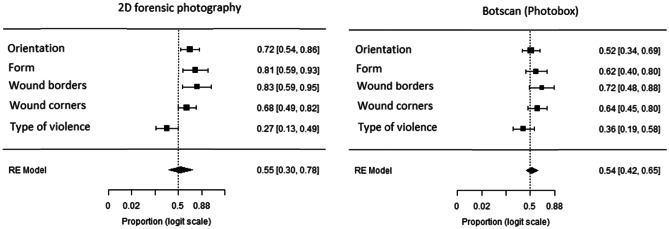
Two examples of our forest-plot analysis for the Photobox results; the complete analysis can be found in the Appendix

We performed a forest-plot analysis to quantify the significant differences between the direct examination and the ones using the technical display methods. An example of this statistical analysis can be found in Fig. 2.

We summarized the statistical analysis in color-coded Table 6.

**Table 6:**
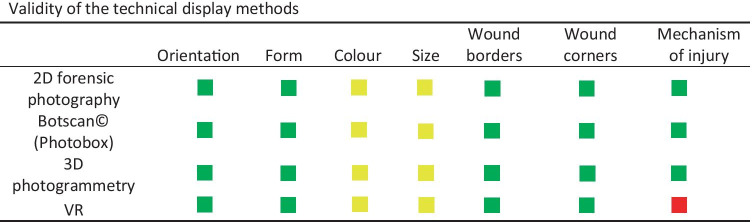
Color-coded summary of our statistical analysis. Green: there is no significant statistical difference between the examination performed directly on the mannequin or in the display method. Yellow: a statistical conclusion cannot be made. Red: the documentation of the category in the technical display method is less accurate than that obtained by direct documentation

## Discussion

Our goal was to compare the real-life examination and interpretation of injuries with technically assisted methods in 2D both on screen and in VR. Our data show no significant differences between any of the technical display methods and the direct examination. However, the specific analysis of the documentation categories shows a more diverse picture. The two categories of *color* and *size* could not be soundly evaluated due to the few identically documented items obtained in the direct evaluation. For the category of *size*, one reason for the low agreement value is that the length and width of the injury stickers were estimated using a ruler and not precisely measured. Unclear wound borders, together with different underlining skin tones, make a consistent demarcation difficult and probably accentuate the interpersonal differences in the estimation of an injury’s size. We have shown in previous publications [11, 15] that precise measurements using technical assisted methods are possible and achieve higher accuracy with the photogrammetry technique than with forensic 2D photography. For the category of *color*, the linguistic variety used in the description, as well as the different number of words used to describe a wound color, was probably the reason for this low level of consensus. This can be attributed to differences in the lexicon and ability to differentiate colors by the involved pathologists [16].

All the categories showed no significant differences with respect to the real-life examination, except the category of suspected *mechanism of injury,* which in our setup was less accurate using the VR method. It is conceivable that the image quality of the HMD was not high enough to allow a clear interpretation of this category. In addition, the residents had no prior training in navigation with VR; therefore, difficulties in moving closer to the injury or with the zooming function could have been a disadvantage compared to the other visualization methods.

There are some practical implications for the forensic documentation process based on our results. It has not yet been established in the forensic field to directly document wounds with pictures from a Botscan© photography set. For this reason, to preserve the spatial geometry, the generation of a 3D model usually follows photography with Photobox. Our results show that Photobox documentation can be used directly for the documentation process of superficial wounds, whether on the 2D photographs acquired by Photobox or the 3D model created based on these data. Furthermore, due to the time-consuming documentation process for forensic 2D photography, the variable possible quality of those pictures, and the inter-user variability, Photobox technology might be a better documentation method for standardizing the documentation and examination procedures and allowing for equal examination results, especially for the documentation of multiple superficial wounds occurring simultaneously in one individual. Regardless of the specific wound constellation, we recommend using an automated photographical documentation process in order to lower the photo-documentation subjectivity. In the future, it could also be possible to combine the Photobox technique with machine learning-based image analysis approaches, for example, as a sorting algorithm to detect relevant pictures prior to expert documentation or to preclassify injuries and allow forensic pathologists to then confirm or deny automatically performed examinations. However, one important prerequisite for any application of Photobox remains that the person pictured needs to be able to stand in an upright position, which limits the usage of Photobox on severely injured individuals.

Our setup had other relevant limitations. The documented wounds were injury stickers that lacked any profile or depth information, thereby making the differentiation between superficial and deep cuts difficult in the real-life examinations. Nevertheless, we decided to establish the real life as our gold standard. The number of forensic pathologists who participated in the current study was also rather low, with only two board-certified pathologists taking part in the real-life examination and four residents using the visualization methods. Furthermore, the influence of the different amount of experience on the documentation of superficial wounds between the residents on the one hand and between the residents and the senior pathologists on the other hand should be taken into consideration. To further validate our findings, future studies should compare examinations performed by forensic pathologists who have similar levels of experience. However, as the findings showed that even less-experienced residents can come to the same conclusion as those obtained by experienced pathologists, we can state that all four methods used in our setup allow for examinations that are as good as those that are directly performed on a person’s body. It is also worth mentioning that, apart from 2D forensic photography, all the technical display methods were new for the participating residents. This difference in routine could have influenced the documentation outcome in disfavor of the other three technical display methods. A final limitation was that some of the subcategories were described using a high linguistic variety because there was only partly standardized jargon available for their description, and this terminology varies across the different German-speaking regions of Switzerland, Germany and Austria. In our setup, the two board certified pathologists were trained in different institutions and even different countries, whereas the residents may have used a more homologous terminology due to their training being within the same institution under the same supervision. These factors reduced the number of *identical* items in the categories of *color* and *size* to a point where a statistical analysis could not yield significant results. Finally, for the other categories, the main investigator of this study was required to group certain descriptions together to make a statistical analysis possible.

In total, the four methods based on photographic documentation procedures showed no significant differences from the examinations performed directly on the mannequin. However, we observed a high level of variability in the inter-reader agreement in the real-life examination, particularly in regard to the subcategories that allowed a high level of linguistic variation during the descriptions. Our findings suggest that the documentation and visualization methods used herein qualify for the examination of superficial wounds. Further research, especially with real injuries, is needed to classify the ideal circumstances in which a technical display method could complement the current standards of forensic evidence documentation, examination and interpretation.

## Conclusion

Technically assisted visualization and documentation options could have sound entry into the forensic field. While VR techniques might need further technical refinement to be used for the examination of superficial wounds, simultaneous photographic documentation using Photobox and 3D photogrammetry models can supplement the current standard approaches in the forensic field.

## Key points

1. Photobox and 3D photogrammetry models can support the forensic documentation process.
2. Technical display methods can be used for the documentation and interpretation of superficial wounds.
3. Wound documentation tends to be described with a high linguistic variability between forensic specialists.

## Supplementary Information

Below is the link to the electronic supplementary material.

Supplementary file1 (PDF 959 KB)
