# Supplementary material for: Comparison of superficial wound documentation using 2D forensic photography, 3D photogrammetry, Botscan© and VR with real-life examination
Source: Forensic Sci Med Pathol. 2021 Aug 18;17(3):422–30. doi: 10.1007/s12024-021-00393-x (PMC8413216; doi:10.1007/s12024-021-00393-x)
Supplement: Supplementary file 1 — Supplementary file1 (PDF 959 KB) [file 12024_2021_393_MOESM1_ESM.pdf]

Appendix

Item sheet used for the documentations, an English translation is available on request

AUSWAHLBLATT

Befundbeschreibung

Orientierung: In Körperlängsachse (KL) | Quer zur Körperlängsachse (KQ)

In Armlängsachse (AL)	Quer zur Armlängsachse (AQ)
In Beinlängsachse (BL)	Quer zur Beinlängsachse (BQ)
Fusswärts geöffnet (FG)	Rumpfseitig oben offen (ROO)
Oben-hinten nach unten vorne (OHUV)	
Rechts oben nach links unten (ROLU)	
Links oben nach rechts unten (LORU)	
Oben aussen unten innen (OAUI)	
Oben innen unten aussen (OIUA)	
Andere Orientierung (AO)	

Bezeichne nicht AO wenn annähernd andere Orientierung, sondern vermerke „XY annh“

Form:			
	Oval (O)	Rund (R)	Bogenförmig (B)
	Gruppiert (G)	Parallel verlaufend (ll)	Streifig (S)
	Landkartenartig (L)	Punktförmig (P)	Andere (A)
	Spindelförmig (Sd)	Fleckförmig (F)	Wolkenartig (W)
	Länglich (Lg)	Flächig (Fä)	Geometrisch (Go)
	Strichförmig (Sf)		

Farbe:		
	Graublau (1)	Livide (2)
	Grün (3)	Gelb (4)
	Rot (5)	Dunkelrot (9)
	Braunrot (6)	Dunkelbraun (7)
	Braun (8)	Zentral Abgeblasst (10)
	Blaurot (11)	Rosa (12)
	Oragne (13)	Blau (14)

Messende Grösse:
Maximale Breite (B)
Maximale Länge (L)
Geschätzte Tiefe der Hauteröffnung anhand der oberflächlichen Erscheinungsform (T)

Hauteröffnungsränder: Glatt, Scharf (G) Unregelmässig, Unscharf (U)
Beschreibung umgebende Hautareale: Verfärbungen (Vf) Abtragungen (Ab) Hautrötung (Hr)

Hauteröffnungswinkel:			
	Spitz (Sz)	Mit Ausläufer (mA)	Ungerade (U)
	Stumpf (Sp)	Gerade (G)	Nicht beurteilbar (NB)

Wenn morphologisch unterschiedliche Wundränder vorhanden sind präzisieren Sie die Zugehörigkeit

Gewaltform

Scharfe Gewalt (SrG)
Unterkategorie: Schnitt (SrGt) Stich (SrGh)
Stich/Schnittkombination (SrGss)
Wenn keine Unterkategorie beurteilbar, nur mit SrG markieren

Halbscharfe Gewalt (HsG)

Stumpfe Gewalt (SpG)
Unterkategorie: Hautunterblutung (Hu) Hauteinblutung (He)
Hautabschürfung (Ha) Quetsch-Riss-Wunde (QR)
Risswunde (R)
Punktförmige Gewalt (PG)
Thermische Gewalt (TG)

Angaben zur wahrgenommenen Beurteilungssicherheit: von 1 = sehr niedrig bis 10 = sehr hoch

Verglichen mit der in Ihrer Tätigkeit üblichen Befundung,
wie sicher fühlten sie sich bei der Beurteilung der Hautläsionen mit der ___________ Methode?

Full results of the Examination

Direct Wound examination on mannequin

	Orientation	Form	Colour	Size	Wound borders	Wound corners	Mechanism of injury
Identical	69 (29)	50 (21)	19 (8)	11 (5)	43 (18)	67 (28)	52 (22)
Similar	2 (1)	21 (9)	52 (22)	81 (34)	50 (21)	17 (7)	43 (18)
Different	29 (12)	29 (12)	29 (12)	7 (3)	7 (3)	17 (7)	5 (2)
Subjective level of certainty	8	3					

Technical display methods

	Orientation	Form	Colour	Size	Wound borders	Wound corners	Mechanism of injury
2D forensic photography	72 (21)	81 (17)	13 (1)	0 (0)	83 (15)	68 (19)	27 (6)
Botscan© (Photobox)	52 (15)	62 (13)	0 (0)	40 (2)	72 (13)	64 (13)	36 (8)
3D photogrammetry	76 (22)	76 (16)	0 (0)	-	61 (11)	79 (22)	27 (6)
VR	69 (20)	81 (17)	13 (1)	20 (1)	39 (7)	71 (20)	23 (5)

Subjective level of certainty	2D forensic photography	Botscan© (Photobox)	3D photogrammetry	VR
Forensic physician (1,2,3,4)	1,1,1,1	2,2,2,2	4,3,4,4	3,4,3,3

Table 7: Summary of our results. The concordance of the three categories of *identical*, *similar* and *different* in the direct wound documentation and the technical display methods are given in percentages, while the absolute number is shown in parenthesis. We used for the comparison of the technical display methods only the items that were documented identically in the direct wound documentation. The degrees of certainty are shown as a scale value ranging from 1 to 10 for the two direct wound documentations, with 10 being defined as equally certain to a real body documentation. For the technical display methods, the four forensic physicians ranked the used methods on a scale ranging from 1 to 4, with 1 being defined as the most certain method.

3 R code for the statistical analysis

```
library(openxlsx)
library(metafor)

data <- read.xlsx(x=" AW.xlsx","Vergleich nach stat bsp")
data
str(data)

res.1 <- rma(measure="PLO", xi = Zaehler, ni=Nenner, data=data, subset =
visualization == "Klassisch 2D", slab=Parameter)
summary(res.1)

tiff(filename = "plot 2d.tiff")
forest(res.1, atransf = transf.ilogit)
text(0,9,"Klassisch 2D")
dev.off()
res.2 <- rma(measure="PLO", xi = Zaehler, ni=Nenner, data=data, subset =
visualization == "2D Photobox", slab=Parameter)

tiff(filename = "plot 2d PB.tiff")
forest(res.2, atransf = transf.ilogit)
text(0,9,"2D Photobox")
dev.off()
res.3 <- rma(measure="PLO", xi = Zaehler, ni=Nenner, data=data, subset =
visualization == "3D Bildschirm", slab=Parameter)

tiff(filename = "plot 3d.tiff")
forest(res.3, atransf = transf.ilogit)
text(0,8,"3D Bildschirm")
dev.off()
res.4 <- rma(measure="PLO", xi = Zaehler, ni=Nenner, data=data, subset =
visualization == "VR", slab=Parameter)

tiff(filename = "plot vr.tiff")
forest(res.4, atransf = transf.ilogit)
text(-0,9,"VR")

dev.off()
```

Original Forest-plot of the statistical analysis

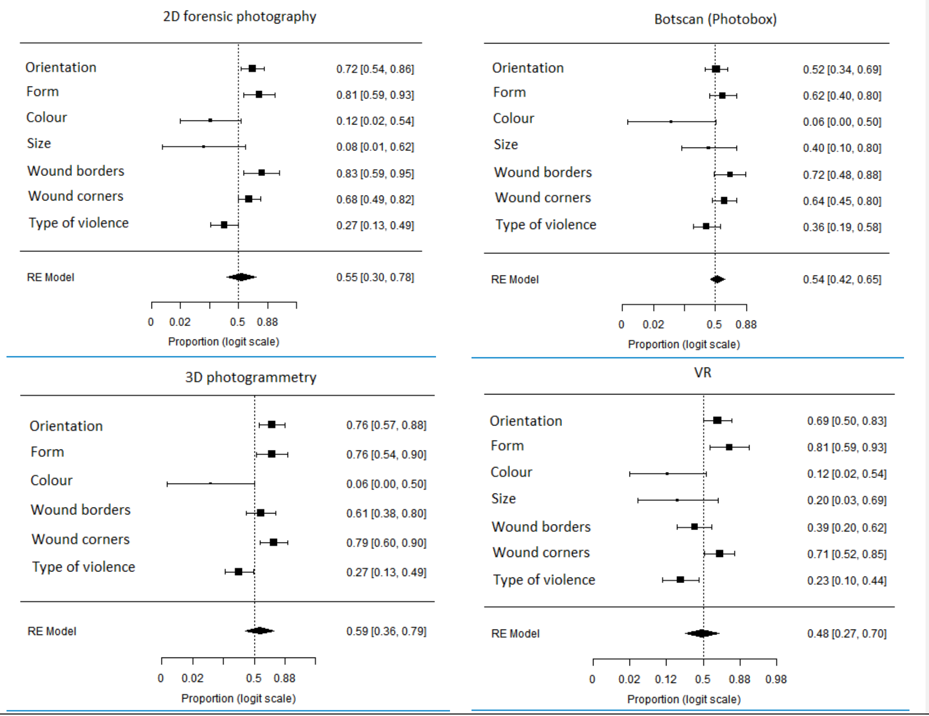

Figure 3: Statistical comparison of the four methods with the direct wound documentation. The black bars stand for the standard deviation of the documented item, and the size of the black squares indicates the weight of the item for the overall comparison. If the final regression model (RE model) crosses the dotted line at 0.5 in the proportion scale (proportion (logit scale)), then there is no significant difference between the outcome of the technical display method and the direct wound documentation. In the article we used the term “mechanism of injury” for the category *type of violence*.
